# A Rare Cause of Prepubertal Gynecomastia: Sertoli Cell Tumor

**DOI:** 10.1155/2015/439239

**Published:** 2015-08-23

**Authors:** Fatma Dursun, Şeyma Meliha Su Dur, Ceyhan Şahin, Heves Kırmızıbekmez, Murat Hakan Karabulut, Asım Yörük

**Affiliations:** ^1^Ümraniye Training and Research Hospital, Pediatric Endocrinology, 34766 Istanbul, Turkey; ^2^Ümraniye Training and Research Hospital, Radiology, Istanbul, Turkey; ^3^Ümraniye Training and Research Hospital, Pediatric Surgery, Istanbul, Turkey; ^4^Ümraniye Training and Research Hospital, Pathology, Istanbul, Turkey; ^5^Göztepe Training and Research Hospital, Pediatric Oncology, Istanbul, Turkey

## Abstract

Prepubertal gynecomastia due to testis tumors is a very rare condition. Nearly 5% of the patients with testicular mass present with gynecomastia. Sertoli cell tumors are sporadic in 60% of the reported cases, while the remaining is a component of multiple neoplasia syndromes such as Peutz-Jeghers syndrome and Carney complex. We present a 4-year-old boy with gynecomastia due to Sertoli cell tumor with no evidence of Peutz-Jeghers syndrome or Carney complex.

## 1. Introduction

Prepubertal gynecomastia is characterized by the presence of palpable unilateral or bilateral breast tissue in boys, without other signs of sexual maturation. Gynecomastia is common in normal males in the neonatal period, at early puberty, and with increasing age [1]. Prepubertal gynecomastia is a rare condition, and there are limited numbers of case reports in the medical literature. Some cases were associated with excessive aromatase activity or estrogen producing adrenal or testis tumors [1].

Sertoli cell tumors (SCTs) account for 2% of prepubertal testicular tumors and very few have occurred in the first decade of life. The most common presenting symptom of a testicular tumor is painless scrotal mass. Gynecomastia can be seen in approximately 5% of patients with testicular mass. Most of the SCTs in prepubertal boys, which are generally bilateral and diffuse, are in the content of Peutz-Jeghers Syndrome (PJS) or Carney complex [2].

## 2. Case Report

**Figure 1:**
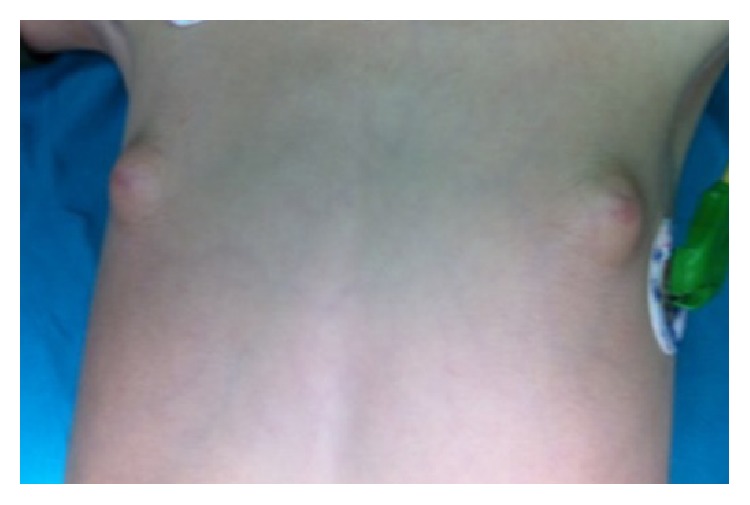
Bilateral gynecomastia.

**Figure 2:**
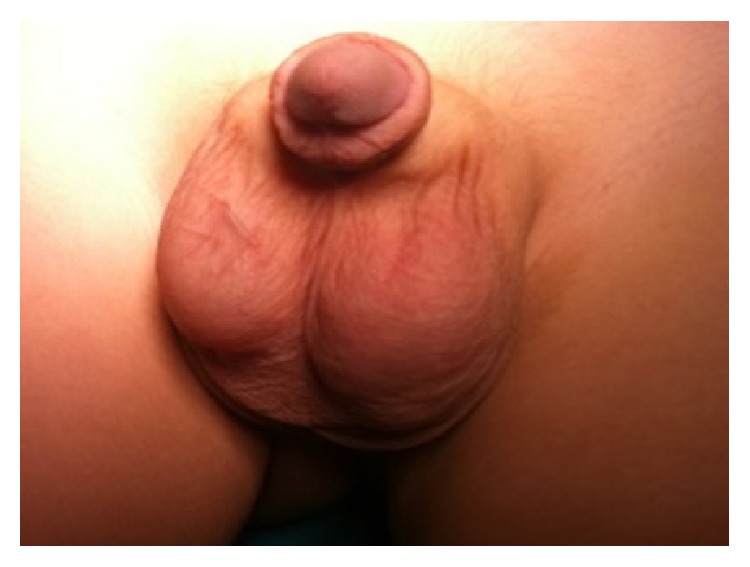
Asymmetrical testicles.

A 4-year-old boy was referred to pediatric endocrinology because of bilateral breast enlargement. There was no history of a chronic disease, medication, or a familial disorder. Height was 114 cm (+1.2 SDS), weight was 20 kg (+0.7 SDS), and physical examination revealed bilateral gynecomastia. Breast development appeared as Tanner Stage-2 (Figure 1), axillary and pubic hair were absent, stretched penile length was 6 × 1.5 cm, and right testis was 2 mL and left testis was 5 mL (Figure 2). Hormone levels were in normal ranges (Table 1); tumor markers were negative while scrotal ultrasonography (USG) exhibited a 8 × 12 mm solid lesion with cystic component in the left testis. The committee on tumoral diseases agreed on the decision to perform a testis-sparing surgery in the light of examination of frozen sections. However the large and cystic mass left no adequate testis tissue to conserve, so a left orchiectomy was performed. Abdomen and thorax Computed Tomography (CT) imaging were normal. Histopathological investigation revealed a SCT which had positive staining with inhibin, vimentin, and calretinin. Gynecomastia regressed at the end of three months following the operation.

## 3. Discussion

Gynecomastia is common in boys at early puberty, while prepubertal gynecomastia is a rare condition. It is generally considered a pathological sign of a possible endocrinopathy, requiring a detailed history, physical examination, and laboratory work-up [1]. Most of the patients have peripubertal gynecomastia (25%) or drug-induced breast development (20%). The frequencies of some remaining causes have been estimated as follows: cirrhosis (8%), primary hypogonadism (8%), testicular tumors (3%), secondary hypogonadism (2%), hyperthyroidism (1.5%), and renal disease (1%). In other cases, prepubertal gynecomastia has been the endocrine manifestation of rare syndromes, such as PJS [3].

The records of Prepubertal Testicular Tumor Registry include forty-two patients with stromal tumors, of whom only 10 patients had SCT with an overall mean age of 52.5 months at presentation [1, 4]. They are sporadic in 60% of the reported cases, but in the remaining cases they are linked to multiple neoplasia syndromes such as PJS and Carney complex [5, 6]. The neoplastic Sertoli cells overexpress p450 aromatase (CYP19A1), which is normally found in only low concentrations in prepubertal Leydig cells. Aromatase allows for increased conversion of 1.4-androstenedione (the major source of androgens from the adrenal gland in prepubertal males with SCTs) to estrone. As only minimal elevations of estrogens are needed to advance the bone maturation and cause gynecomastia, physicians should take into consideration the sensitivity of the assay used to measure estrogens [6]. We could not measure level of serum estrone. Testosterone is also converted to estradiol, but this is less of an issue in prepubertal boys with SCTs [5]. Although these hormone levels may still remain below the detection limit of standard assays, the sensitivity of the growth plates and breast tissue to estrogens may lead to growth acceleration, advanced bone age, and gynecomastia in prepubertal boys. Such presentation is similar to that observed in cases of aromatase excess syndrome due to rearrangements in the CYP19A1 gene [7].

There is no specific differential immunoprofile for this tumor. Vimentin, inhibin, and calretinin seem more widely expressed and may help the diagnosis of SCTs [2]. Malignancy is found in approximately 17% of patients with SCTs. Malignant SCTs usually occur in older patients (mean age 39 years) and those who have unilateral and unifocal disease (for comparison, the mean age of presentation for benign tumors is 17 years). Indices of malignancy include mitotic count greater than 3 per 10 high-power fields, size larger than 4 cm, significant nuclear atypia, tumor necrosis, and angiolymphatic invasion [6, 8, 9]. Our patient had no evidence of malignancy. Gynecomastia has been well reported in prepubertal boys with SCTs but in the context of PJS. The tumors in this context are more diffuse with calcifications, often bilateral, and may respond to medical treatment [7, 10]. SCTs in the context of Carney Complex are similarly diffuse and bilateral [11]. Also our patient had unilateral testicular mass.

In conclusion, our patient had unilateral testicular solid-cystic mass with no evidence of perioral lesions, intestinal symptoms, or family history for syndromes. Although unilateral testicular mass is very rarely reported, this patient should be followed up carefully in terms of other clinical findings of PJS as well as Carney Complex. This case was reported to remind of a very rare condition in the etiology of prepubertal gynecomastia and emphasize on the importance of careful physical examination and scrotal USG.

## Figures and Tables

**Table 1:** Results of laboratory and imaging studies of the patient.

LH^*∗*^ (*N* < 0.05)	0.05 mIU/mL
FSH^*∗*^ (*N*: 0.1–3)	0.11 mIU/mL
Total testosterone (*N*: <0.14)	0.13 ng/mL
TSH^*∗*^ (*N*: 0.7–6.4)	1.02 mIU/mL
Free T4^*∗*^ (*N*: 0.8–2.2)	1.17 ng/dL
17OHP^*∗*^ (*N*: 0.03–0.9)	0.9 ng/mL
Prolactin (*N*: 2.89–35)	23 ng/mL
Cortisol (*N* > 9)	13.4 mcg/dL
DHEA-S^*∗*^ (*N*: 5–57)	17.8 mcg/dL
Estradiol (*N* < 10)	<10 pg/mL
SHBG^*∗*^ (*N*: 11.2–100)	125.3 nmol/L
Bone age	5 years 9 months
Beta-HCG^*∗*^ (*N* < 1)	0.1 mIU/mL
Alpha fetoprotein (*N* < 9)	0.3 ng/mL
Carcinoembryogenic antigen (*N*: 0–3)	0.9 ng/mL
Scrotal ultrasonography	Right testis: 0.5 mL. 12 × 8 mm solid mass with cystic component in the central area was detected in left testis.
Thorax Computed Tomography	Normal
Abdomen Computed Tomography	Normal

^*∗*^LH: luteinizing hormone; FSH: follicle stimulating hormone; TSH: thyroid stimulating hormone; Free T4: free tiroksin-4; 17OHP: 17-hydroxyprogesterone; DHEA-S: dehydroepiandrostenedione-sulphate; SHBG: sex-hormone-binding-globulin; Beta-HCG: beta-human chorionic gonadotropin; *N*: normal range.

## References

[1] Einav-Bachar R., Phillip M., Aurbach-Klipper Y., Lazar L. (2004). Prepubertal gynaecomastia: aetiology, course and outcome. *Clinical Endocrinology*.

[2] Burgu B., Aydoğdu O., Telli O. (2011). An unusual cause of infantile gynecomastia: sertoli cell tumor. *Journal of Pediatric Hematology/Oncology*.

[3] Ferraro G. A., Romano T., De Francesco F. (2013). Management of prepubertal gynecomastia in two monozygotic twins with Peutz-Jeghers syndrome: from aromatase inhibitors to subcutaneous mastectomy. *Aesthetic Plastic Surgery*.

[4] Ross J. H., Kay R. (2004). Prepubertal testis tumors. *Reviews in Urology*.

[5] Brodie A., Inkster S., Yue W. (2001). Aromatase expression in the human male. *Molecular and Cellular Endocrinology*.

[6] Gourgari E., Saloustros E., Stratakis C. A. (2012). Large-cell calcifying Sertoli cell tumors of the testes in pediatrics. *Current Opinion in Pediatrics*.

[7] Crocker M. K., Gourgari E., Lodish M., Stratakis C. A. (2014). Use of aromatase inhibitors in large cell calcifying sertoli cell tumors: effects on gynecomastia, growth velocity, and bone age. *Journal of Clinical Endocrinology and Metabolism*.

[8] Halat S. K., Ponsky L. E., MacLennan G. T. (2007). Large cell calcifying Sertoli cell tumor of testis. *The Journal of Urology*.

[9] Kratzer S. S., Ulbright T. M., Talerman A. (1997). Large cell calcifying sertoli cell tumor of the testis: contrasting features of six malignant and six benign tumors and a review of the literature. *American Journal of Surgical Pathology*.

[10] Ulbright T. M., Amin M. B., Young R. H. (2007). Intratubular large cell hyalinizing sertoli cell neoplasia of the testis: a report of 8 cases of a distinctive lesion of the Peutz-Jeghers syndrome. *The American Journal of Surgical Pathology*.

[11] Brown B., Ram A., Clayton P., Humphrey G. (2007). Conservative management of bilateral sertoli cell tumors of the testicle in association with the carney complex: a case report. *Journal of Pediatric Surgery*.

